# Treatment of minocycline-induced hyperpigmentation with 730 nm Ti:sapphire picosecond laser

**DOI:** 10.1016/j.jdcr.2023.10.022

**Published:** 2023-11-30

**Authors:** Ali Alajmi, Ghassan Niaz, Kachiu Lee, Eric F. Bernstein

**Affiliations:** a Main Line Center for Laser Surgery, Ardmore, Pennsylvania; b Departement of Dermatology, King Faisal Specialist Hospital and Research Centre, Jeddah, Saudi Arabia; c Department of Dermatology, Temple University, Philadelphia, Pennsylvania; d Department of Dermatology, School of Medicine, University of Pennsylvania, Philadelphia, Pennsylvania

**Keywords:** 730-nm Ti:sapphire, hyperpigmentation, laser, minocycline, Nd:YAG laser, picosecond, Q-switched

## Introduction

Minocycline is a broad-spectrum antibiotic agent with an antiinflammatory activity frequently used in dermatology.^1^, ^2^, ^3^, ^4^ The notable efficacy and favorable safety profile of minocycline have made it a preferred choice primarily for treating acne and various other dermatological conditions, often necessitating long-term administration to achieve optimal outcomes.^4^

Despite the manifold advantages of minocycline therapy, the emergence of cutaneous hyperpigmentation as an infrequent yet consequential adverse effect poses a distinct challenge. This unwanted pigmentation is a well-documented, dose-dependent side-effect appearing most commonly in sun-exposed areas, mucosa, teeth, and sclera. The pathophysiologic causes of this hyperpigmentation are believed to result from its metabolites being deposited in the dermis of affected skin, and inflammation causing epidermal melanin to also be deposited in the dermis. The chronic photoactivation of these constituents likely contributes to the formation of this drug-induced pigmentation cascade.^5^

We present a case series of patients with minocycline-induced skin pigmentation treated with a both nanosecond-domain and picosecond-domain lasers using a variety of wavelengths. Finding the optimal combination of lasers and treatment parameters often requires multiple test spots, and changes to the treatment regimen once pigmentation response reaches a plateau with a given device or treatment regimen. Furthermore, a noticeable adjustment in the wavelength will be observed during the subsequent treatment sessions, with the intention of maximizing efficacy.

## Patient 1

**Fig 1:**
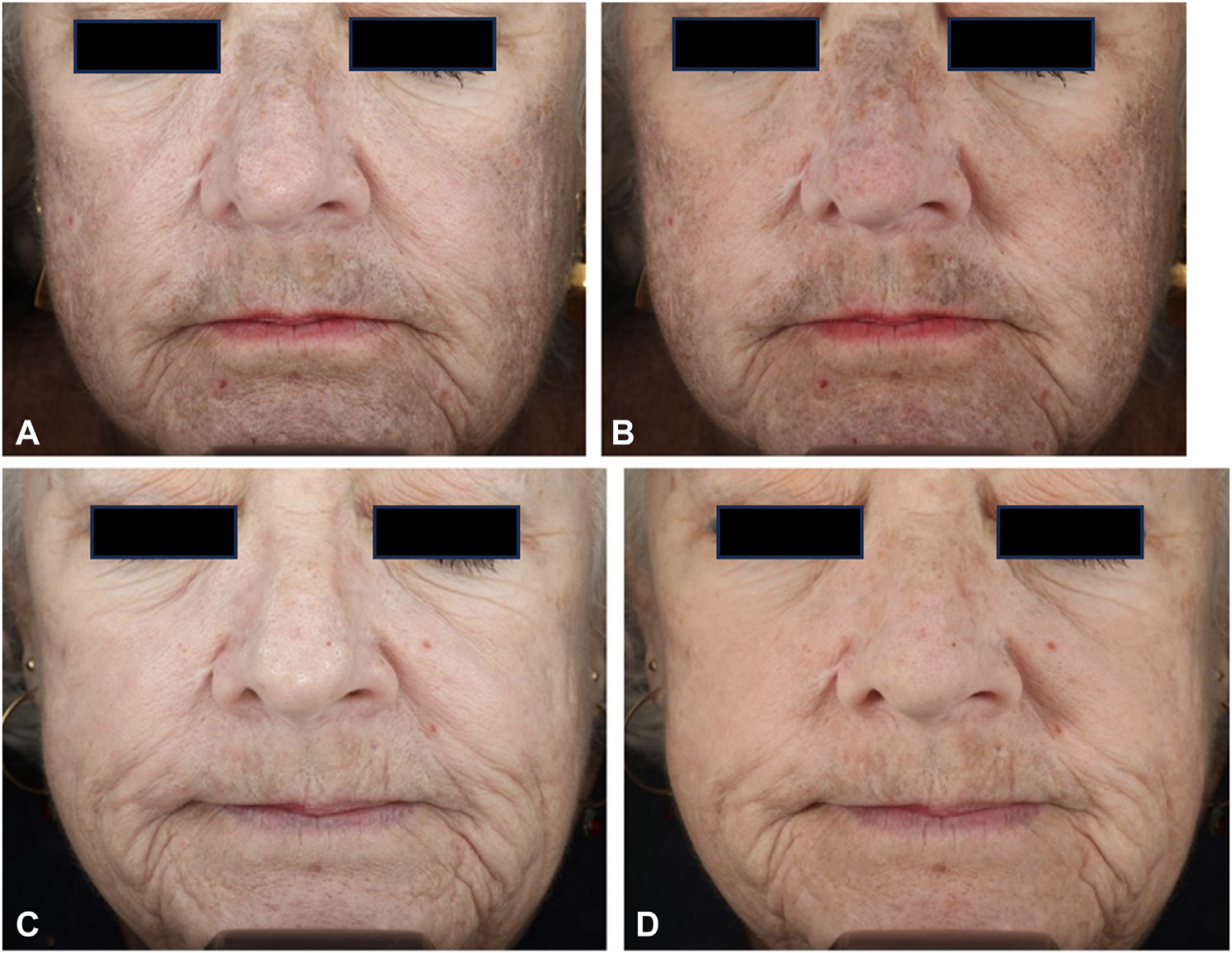
Front view of the patient’s face showing **(A)** nonpolarized and **(B)** cross-polarized photography before laser treatment demonstrates classic slate-gray hyperpigmentation of characteristic minocycline pigmentation. On the lower pictures, front view of the patient showing **(C)** nonpolarized and **(D)** cross-polarized photography of the patient’s face after completion of 3 treatments with 730 nm picosecond laser.

A 71-year-old woman with Fitzpatrick skin type I and a history of acne scaring presented to our clinic with gradual onset of diffuse, brown, symmetric hyperpigmentation predominantly in the sun-exposed areas her face, developing over the course of 2 to 3 years. She had been taking minocycline to treat acne vulgaris for 30 years before her presentation, with a daily dose of 100 mg. One year before presenting to our practice, her minocycline treatment has been stopped, and the patient underwent a solitary 1064 nm neodymium:yttrium-aluminum-garnet (Nd:YAG) Q-switched laser procedure at an external facility, but no noticeable improvement was observed. On examination, light brown and slate-gray hyperpigmented patches were predominantly observed on the temples, nose, chin, cutaneous lips, and lateral cheeks, without involvement of the mucosa or nails (Fig 1). Based on the patient’s medication history and clinical examination findings, the patient was diagnosed with minocycline-induced hyperpigmentation.

**Fig 2:**
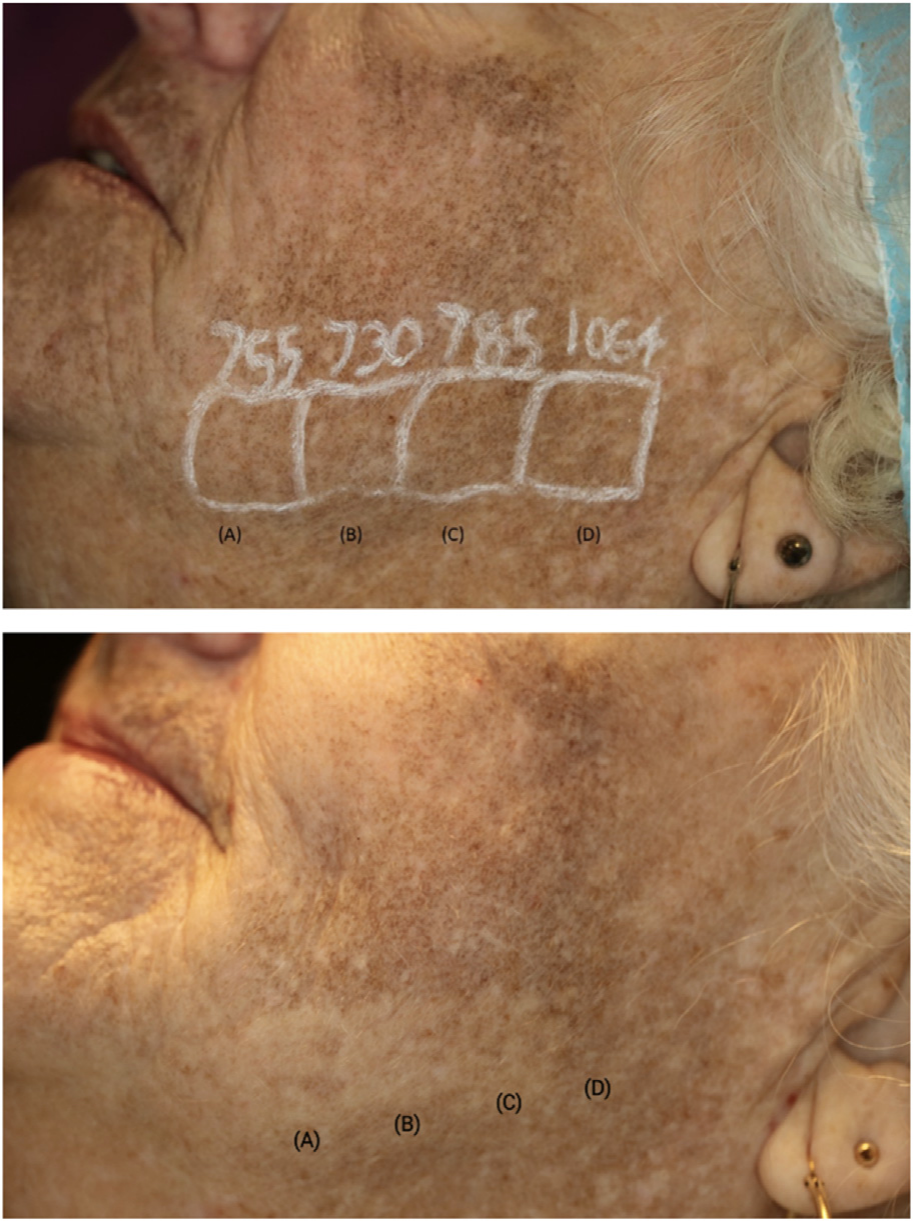
The upper picture showing different test spots with **(A)** 6.4 J/cm^2^ fluence, 755 nm/2 mm spot size, **(B)** 1.8 J/cm^2^ fluence, 730 nm and 3 mm spot size, **(C)** 1.4 J/ cm^2^ fluence, 785 nm and 3 mm spot size, and **(D)** 3.2 J/cm^2^ fluence, 1064 nm and 4 mm spot size. The lower picture showing tested areas with most response to 730 nm **(B)** followed by 755 nm **(A)** picosecond laser after 4 weeks.

To ascertain the optimal treatment parameters for this patient, test spots using 4 different lasers were performed. Because the patient reported that they had no improvement after treatment with a 1064-nm, nanosecond-domain laser and so many other options were available, this laser was not included in the test spots. Test spots were administered with: a picosecond-domain 1064 nm laser (PicoWay, Candela Medical Corporation) using 3.2 J/cm^2^ with a 4-mm spot, a laser-pumped Ti:sapphire picosecond-domain laser emitting 730-nm (PicoWay, Candela Medical Corporation) using 1.8 J/cm^2^ and a 3 mm spot, a 755-nm alexandrite picosecond-domain laser (PicoSure, Cynosure, LLC) using 6.4 J/cm^2^ fluence and a 2 mm spot, and a 785-nm Ti:sapphire picosecond-domain laser (PicoWay, Candela Medical Corporation) using 1.4 J/cm^2^ and a 3 mm spot (Fig 2). The efficacy of the test spots was evaluated 1 month after treatment, and the test site treated the 730 nm, Ti:sapphire laser-pumped-laser handpiece with a fluence of 1.8 J/cm^2^, and a 3 mm spot exhibited the most favorable response (Fig 2). Following administration of 4% topical lidocaine cream (LMX4, Ferndale Laboratories Inc Company) for 45 minutes, the areas of the face evidencing minocycline pigmentation were treated with a single pass the 730 nm, Ti:sapphire laser with a fluence of 1 J/cm^2^ and a 4 mm spot. Immediate white frosting of the pigmented areas was observed as the desired end point. Immediately after treatment, the treated areas demonstrated transient erythema and mild edema, which improved within a few hours after treatment by patient report. Three treatments were administered at 6 to 8 week intervals resulting in pigment clearance (Fig 1).

## Patient 2

**Fig 3:**
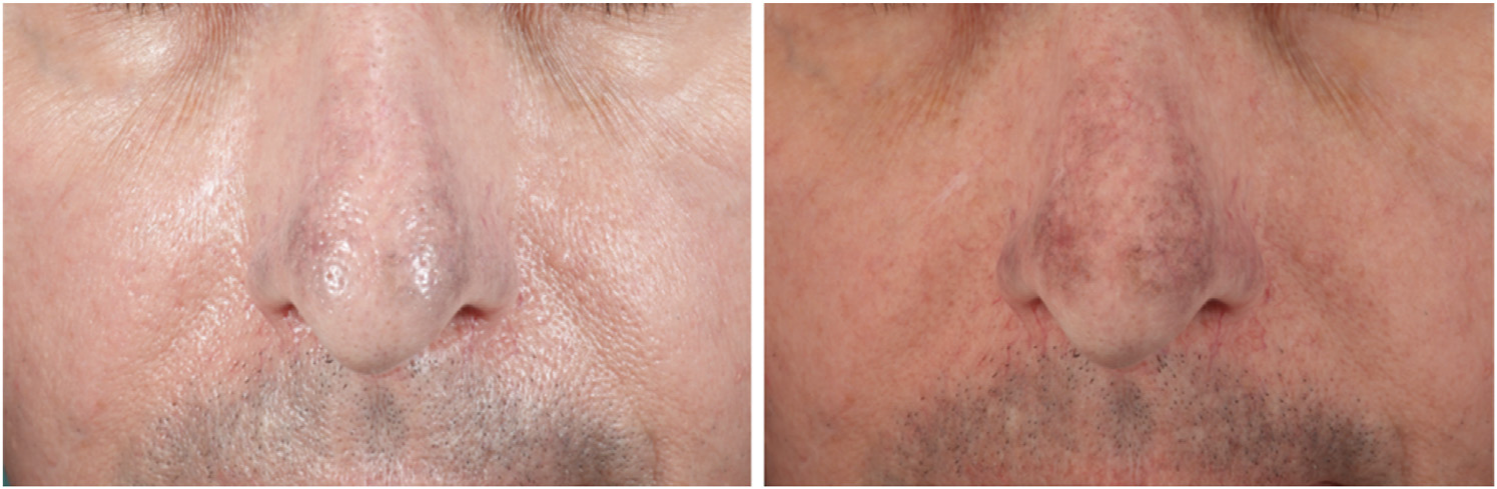
Front view of the patient’s face—nonpolarized (*left*) and cross-polarized (*right*) photography before laser treatment demonstrates slate-gray hyperpigmentation on the nose and upper cutaneous lip.

**Fig 4:**
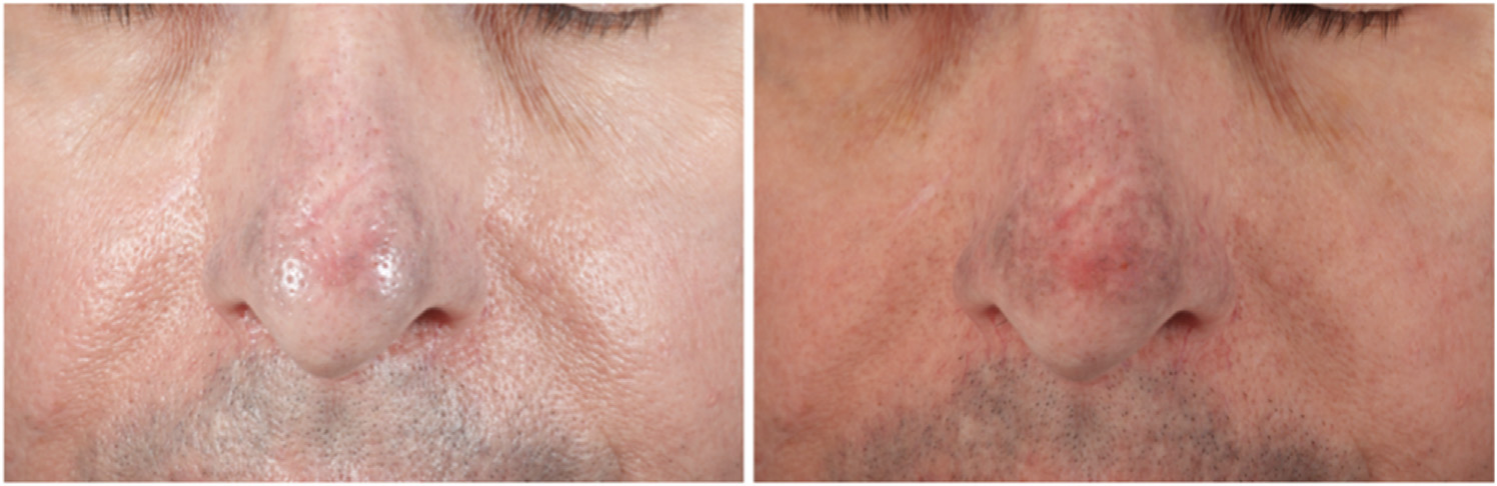
Front view of the patient’s face—nonpolarized (*left*) and cross-polarized (*right*) photography, 2 months later after 1 treatment with Nd:YAG Q-switched laser demonstrates minimal response. *Nd:YAG*, neodymium:yttrium-aluminum-garnet.

**Fig 5:**
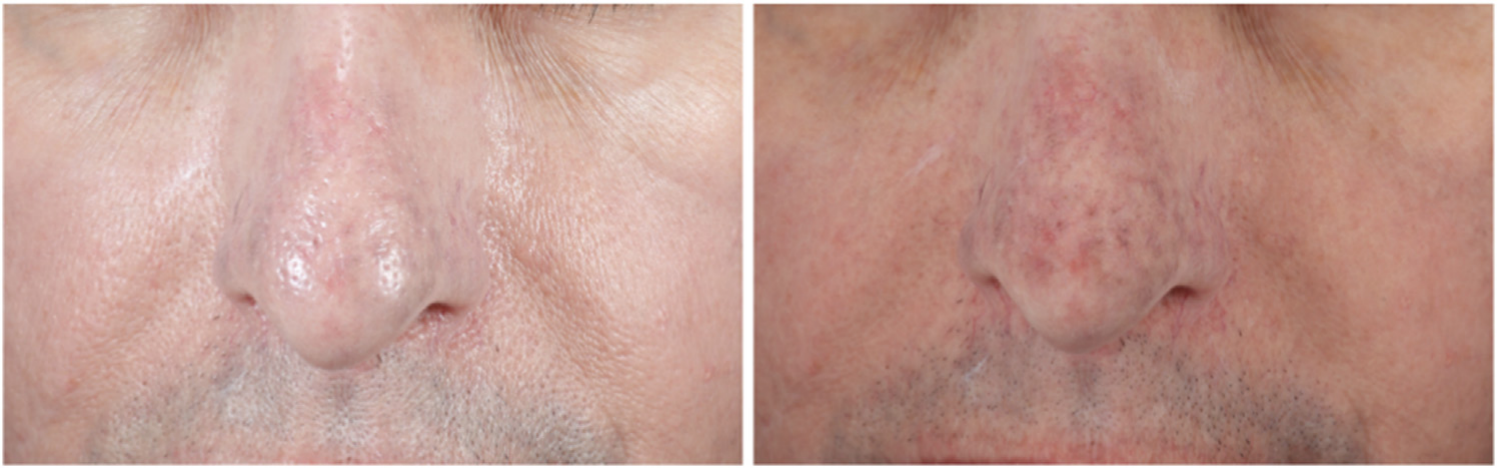
Front view of the patient’s face—nonpolarized (*left*) and cross-polarized (*right*) photography, 2 months later after 1 treatment with a 785 nm picosecond laser demonstrates a good response.

**Fig 6:**
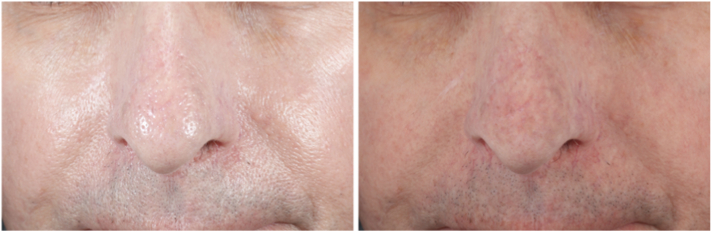
Front view of the patient’s face—nonpolarized (*left*) and cross-polarized (*right*) photography, 2 months later after 1 treatment with a 730 nm picosecond laser demonstrates maximum response.

A 72-year-old man with Fitzpatrick skin type II, who had been taking minocycline intermittently for decades to treat rosacea, presented for treatment of progressive darkening of the central face. The patient had been taking a daily dose of 100 mg of minocycline during rosacea flare-ups for over 10 years, followed by 3 days per week as maintenance therapy for the next 20 years. Approximately 1 year before presentation, the patient noticed a gradual gray discoloration of his nose and perioral region, and was advised by his dermatologist to discontinue his minocycline. On presentation, the patient was examined using a cross-polarized, magnifying headlamp (v600, Syris Scientific), revealing slate-gray pigmentation involving the nose and upper cutaneous lip, consistent with minocycline pigmentation (Fig 3). The oral mucosa and nails, were not involved. The patient was taking no other medications. Following administration of topical 4% lidocaine cream (LMX4, Ferndale Laboratories Inc Company) the patient was treated with a nanosecond-domain, Q-switched 1064 nm laser with a fluence of 6.6 J/cm^2^, and a 5 mm spot size, with minimal improvement noted 2 months after treatment (Fig 4). For his second treatment, a picosecond-domain, Ti:sapphire, 785 nm laser (PicoWay, Candela Corporation), was selected using a fluence of 0.8 J/cm^2^, and a spot size of 4 mm on the nose, and 1.4 J/cm^2^ with a of 3 mm spot on the upper lip. Significant improvement was seen 2 months after this treatment (Fig 5). Two months later a second treatment was administered using the Ti:sapphire, 730 nm, picosecond laser (PicoWay, Candela Corporation) with a fluence of 1.8 J/cm^2^ and a 3 mm spot, resulting in dramatic clearing of the pigment 5 months after his final treatment (Fig 6).

## Patient 3

**Fig 7:**
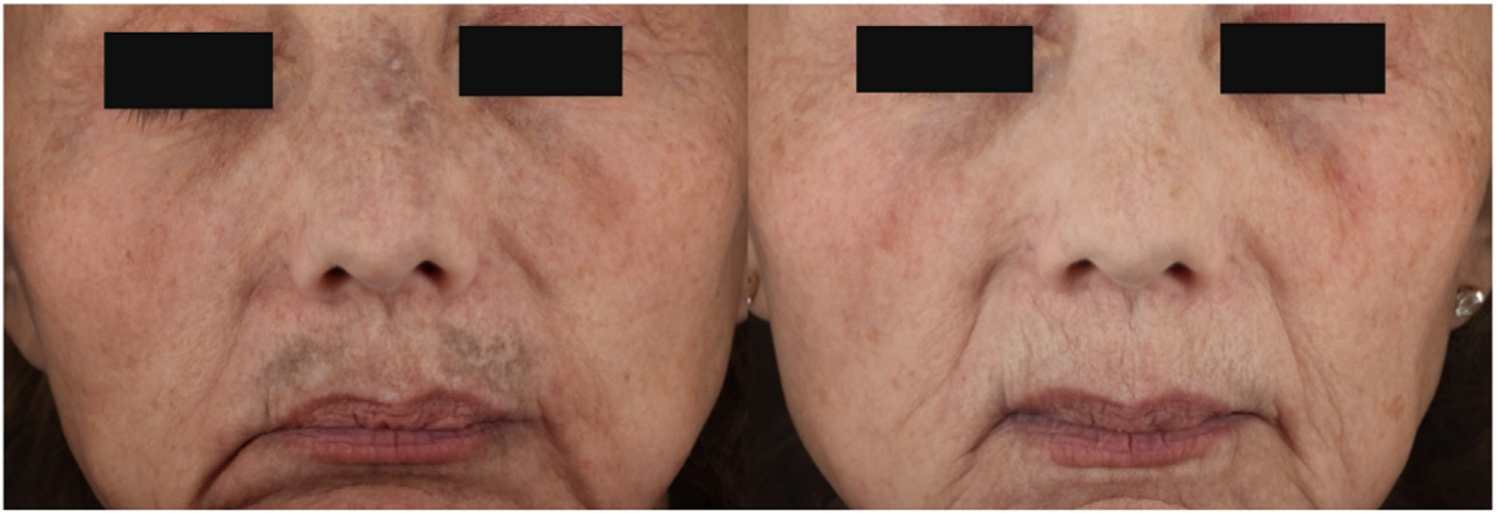
Front view of the patient’s face—cross-polarized photography before (*left*) and after 3 treatments (*right*) with a 1064 nm Q-switched laser.

A 66-year-old woman with Fitzpatrick skin type II presented with a prolonged history of minocycline usage, resulting in pigmentation affecting the nose and both upper and lower cutaneous lips. Notably, minocycline has been discontinued several months before her initial consultation at our facility. She was treated to all areas of pigmentation on her face with a 1064 nm, Q-switched Nd:YAG laser (Con-Bio RevLite SI, Cynosure, Inc) using a fluence of 6 J/cm^2^ and a 4 mm spot. Three months later, a second treatment was administered using 7 J/cm^2^ for the nose and upper cutaneous lip, and 4 J/cm^2^ on the lower cutaneous lip, with a 4 mm spot. Four months later, the patient received a third treatment with the same device using 8 J/cm^2^ and a 4 mm spot. With decreasing pigmentation after each treatment, the subsequent treatments require using a higher fluence to achieve the same tissue end point of immediate whitening. Five-years after the final treatment the patient presented for treatment of an unrelated condition and demonstrated long-term improvement (Fig 7).

## Discussion

Drug-induced hyperpigmentation has been representing 10% to 20% of reported cases of acquired hyperpigmentation.^5^ Among the various medications associated with the development of drug-induced hyperpigmentation, minocycline is frequently reported as an agent. However, it is important to note that only up to 15% of patients using minocycline may experience pigmentation issues.^2^^,^^5^

**Fig 8:**
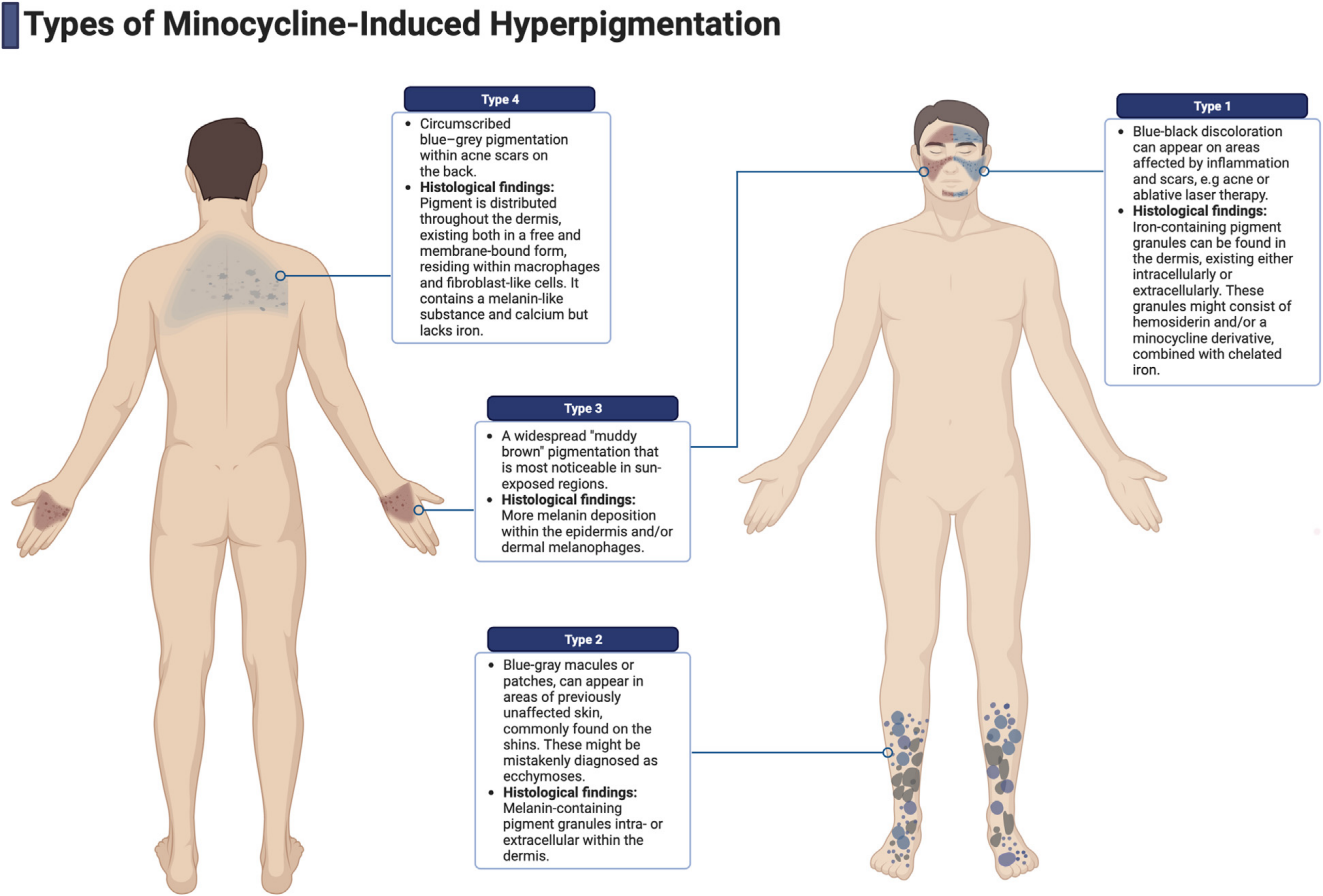
Diagram demonstrating the typical location for the 4 types of hyperpigmentation associated with minocycline.

Minocycline-induced pigmentation (MIP) can manifest as a consequence of long-term minocycline therapy, believed to stem from the deposition of insoluble minocycline complexes, pigmented or reactive metabolites, elevated melanin levels, or a combination thereof.^4^ Among the 4 recognized types of MIP, type I is the most prevalent and is characterized by the presence of blue-black macules primarily within the scarring regions, notably acne scars, predominantly located on the face (Fig 8). This type is postulated to result from the formation of iron chelates involving minocycline. In contrast, type III is the least common variant and manifests as a muddy brown pigmentation known as the “dirty skin syndrome” within sun-exposed areas, typically observed on the face. This type is thought to arise from heightened melanin levels in the epidermis and dermal macrophages. Notably, a newly reported type of pigmentation, type IV, shares similarities with type III but is limited to scars, encompassing both sun-exposed and protected areas of the body.^2^^,^^3^^,^^6^^,^^7^

The initial approach to managing drug-induced hyperpigmentation involves discontinuation of the causative agent and exploration of alternative treatment options, aiming to partially or completely resolve this complication. Historically, quality-switched lasers such as Alexandrite, Ruby, and Nd:YAG nanosecond lasers have been the favored modality for addressing this condition.^8^ We hold the belief that both nanosecond and picosecond-domain lasers demonstrate effective results in addressing drug-induced pigmentation. Although they have proven their effectiveness in managing hyperpigmentation, it is important to acknowledge that pigments resulting from the same drug can exhibit varying responses in different patients. In comparison to picosecond lasers, Q-switched lasers, sometimes, might have certain limitations, as they frequently necessitate multiple treatment sessions and could lead to incomplete outcomes.^2^^,^^4^^,^^7^, ^8^, ^9^, ^10^

The exact mechanism behind the efficacy of picosecond lasers in treating minocycline pigmentation remains poorly understood. However, one proposed hypothesis for their superiority over Q-switched lasers is the significantly shorter pulse duration, which is less than the thermal relaxation time of the target chromophore. This same mechanism also accounts for the enhanced response of tattoo ink as a target chromophore to picosecond lasers when compared with Q-switched lasers. Although Q-switched lasers deliver energy to a target chromophore in nanoseconds (1 billionth of a second), picosecond lasers do so in picoseconds (1 trillionth of a second). The shorter pulse durations may create a variance in photoacoustic effect in addition to the photothermal effect. For selective photoacoustolysis to take place, the pulse duration must be equal to or less than the acoustic diffusion time of the target chromophore, representing another potential mechanism through which picosecond lasers may exhibit greater efficacy with specific chromophores compared with nanosecond lasers.^9^ Within our case series, the first patient exhibited the most pronounced response to the 730 nm wavelength compared with the other wavelengths tested. The 730 nm, 246 ps Ti:sapphire lasers on the market emit picosecond-domain pulses and offer a peak power of 0.41 GW. This short pulse duration is advantageous for targeting smaller cytoplasmic granules while minimizing photothermal effects, which may contribute to a decreased risk of adverse effects.^8^ Moreover, the superior response observed with the 730 nm wavelength, in contrast to other wavelengths employed in our case, may be attributed to its higher peak power or its ability to effectively target chromophores at appropriate depths within the dermis and basement membrane of the epidermis.^11^

In this case series, we have observed an inconsequential response to the implementation of the Q-switched laser in the 2 patients under investigation. However, it is crucial to highlight that a subset of patients with MIP, as documented in existing literature, has demonstrated remarkable outcomes when treated with the Q-switched Nd:YAG laser.^2^ The efficacy of this treatment approach could be attributed to various factors, including the depth and density of the deposited pigments, the individual’s unique skin reactivity to the medication or its metabolites, and other incompletely understood mechanisms. We believe based on our experience that switching devices can result in faster clearance once maximal fluences, wavelengths and pulse durations reached with a given device.

## Conclusion

Our case series presents the first documented instance of successful treatment of minocycline-induced hyperpigmentation utilizing a 730 nm wavelength Ti:sapphire picosecond-domain laser. This remarkable finding highlights the potential significance of picosecond-domain lasers as a valuable addition to the therapeutic armamentarium for managing challenging and recalcitrant cases of cutaneous hyperpigmentation arising as an adverse effect of medication, such as MIP. Furthermore, the utilization of the 730 nm wavelength in our cases, which emits the shortest picosecond-domain pulse currently available in the market, highlights the potential benefits of ultrashort pulse durations in achieving superior outcomes with minimal photothermal effects. Remarkably, certain patients exhibit diverse responses to varying wavelengths and pulse durations. Intriguingly, the Q-switched laser, characterized by its nanosecond pulse duration, has demonstrated comparable efficacy to picosecond lasers in certain cases, albeit the reasons behind this phenomenon remain elusive. Continued research and investigation are warranted to optimize treatment protocols further, deepen our understanding of the underlying mechanisms, and refine patient selection criteria. This endeavor will lead to the provision of more effective and personalized interventions for resolving minocycline-induced hyperpigmentation, empowering dermatologists to offer their patients improved outcomes and enhanced well-being.

## Conflicts of interest

None disclosed.
